# Radiotherapy elicits immunogenic cell death and metabolic shifts in the tumor microenvironment: implications for immunotherapy

**DOI:** 10.7150/ijms.109515

**Published:** 2025-07-11

**Authors:** Zi-Shan Hang, Yue-Ying Huang, An Song, Zhi-Jun Sun

**Affiliations:** 1 State Key Laboratory of Oral & Maxillofacial Reconstruction and Regeneration, Key Laboratory of Oral Biomedicine Ministry of Education, Hubei Key Laboratory of Stomatology, School & Hospital of Stomatology, Frontier Science Center for Immunology and Metabolism, Taikang Center for Life and Medical Sciences, Wuhan University, Wuhan, China.; 2 The Affiliated Stomatological Hospital, State Key Laboratory Cultivation Base of Research, Prevention and Treatment of Oral Diseases, Jiangsu Province Engineering Research Center of Stomatological Translational Medicine, Nanjing Medical University, Nanjing, China.

**Keywords:** radiotherapy, tumor microenvironment, immunogenic cell death, metabolism, immunotherapy

## Abstract

Radiotherapy, one of the most utilized strategies to combat malignancies, has been constantly explored for its effectiveness and optimized, and it is currently operating at the molecular level. The tumor microenvironment (TME), where complicated changes take place under radiotherapy and other treatments, inevitably draws our attention to metabolic alterations, immunogenic cell death (ICD) and immunological interactions. In response to radiotherapy, tumor metabolism promotes DNA and membrane repair processes and reduces oxidative stress, thereby collectively alleviating the occurrence of cell death. Moreover, the induction of pyroptosis, necroptosis and ferroptosis under radiotherapy has the potential to increase antitumor immunity. Therefore, comprehensive knowledge about how radiotherapy triggers these modalities of ICD mechanically is necessary for developing nanomedicines with more accurate targets. In addition, information on clinical advancements as well as the management of adverse events is important for investigating radiotherapy combined with immunotherapy. This review provides an overview of up-to-date findings on metabolic changes and ICD under radiotherapy and provides insight into the status of the TME.

## 1. Introduction

Radiation therapy stands as a cornerstone of cancer treatment, with estimates suggesting that approximately half to two-thirds of oncologic patients receive radiotherapy at some stage in their treatment, either as a monotherapy or in conjunction with surgery, chemotherapy, or immunotherapy 1. However, although radiotherapy is effective in eradicating tumor cells, it also induces alterations in tumor metabolism and the tumor microenvironment (TME). The TME is classified into three different immunophenotypes, namely, immune-excluded, immune-desert, and immune-inflamed phenotypes 2. In immune-excluded tumors, CD8^+^ T cells are localized at malignancy margins and fail to infiltrate the main tumor mass, whereas immune-desert tumors lack CD8^+^ T cells within the tumor and its surrounding periphery. Both phenotypes are regarded as “cold” because of their abundance in immunosuppressive cells and lack of immunogenicity. In contrast, immune-flamed tumors, described as “hot” tumors, exhibit high T-cell infiltration, high PD-L1 expression and high tumor mutational burden. “Hot” tumors are more responsive to immune checkpoint blockades (ICBs) than “cold” tumors are, which indicates that turning “cold” tumors “hot” can be crucial to boosting immunotherapy 3.

Postradiotherapy, tumors exhibit significant TME and metabolic modifications. Initially, the metabolism of tumor cells is modified to mitigate their cytotoxic effects, and these changes extend to the TME *via* metabolite secretion 4. In addition, various nonneoplastic stromal cells within the TME are directly impacted by radiotherapy, leading to significant metabolic alterations. Alterations in the physical and chemical properties of the TME subsequently influence metabolic pathways. These metabolic reprogramming processes play pivotal roles in facilitating energy utilization, mitigating oxidative damage, and repairing DNA and the cellular membrane 5, 6. This understanding allows for the design of metabolic inhibitors that target these modified sites, and there have been relevant reviews summarizing drugs that target tumor metabolism 6, 7. Certain metabolic drugs that target tumors have demonstrated unique mechanisms of inducing cell death 8. When single or combined cancer metabolic therapies fail, inducing cell death *via* the combination of radiotherapy and immunotherapy can be promising.

Radiotherapy induces cell death that is immunogenic and proinflammatory, including pyroptosis, necroptosis and ferroptosis, which can be an effective way to turn “cold” tumors “hot” and prepare the TME for immunotherapy. Pyroptosis is morphologically characterized by nuclear condensation, cell swelling and pore formation on the cell membrane 9. Mechanistically, several pathways of pyroptosis can be initiated by stress, including radiotherapy, and lead to pore formation by different gasdermins *via* different cascades 10. Necroptosis, perceived as regulated necrosis due to its morphological similarity to necrosis, is an indispensable part of cell death under radiotherapy 9. Necroptosis exhibits immunogenic and proinflammatory properties and is partially responsible for tumor metastasis 11. Ferroptosis, an iron- and lipid-dependent cell death modality, has been defined and molecularly described in the last decade 12. The mitochondrion, as the key organelle involved in ferroptosis, is not only closely related to reactive oxygen species (ROS) generation and lipid metabolism mechanistically but also shrinks with membrane densification and cristae disappearance morphologically 9. Currently, ferroptosis stands at the intersection of metabolic modulation and cell death induction in nanomedicine development.

This review focuses on the radiotherapy-induced TME, metabolic changes and cell death. Within the TME, complicated interactions triggered by radiotherapy are elucidated, which can enhance our understanding of the multitarget effects of cancer treatment and optimize its application in reshaping an inflammatory environment conducive to immunotherapy. Specifically, alterations in metabolic pathways, including glycolysis, glutaminolysis and lipid metabolism, are discussed. In addition to elucidating the relationship between altered metabolism and cell death, attention is given to radiotherapy-induced pyroptosis, necroptosis and ferroptosis at the molecular mechanism level, revealing potential nanomedicine targets. Additionally, the modulatory effects of these immunogenic cell death (ICD) modalities on antitumor immunity are described. From a clinical perspective, advancements in radiotherapy combined with immunotherapy are discussed. Furthermore, adverse events of radiotherapy have been noted, and preventive strategies have been explored to better synergize with immunotherapy and ultimately achieve a more satisfactory outcome. Radiotherapy, as a traditional treatment modality, presents opportunities and challenges when combined with other treatments, especially immunotherapy.

## 2. Radiotherapy modulates the TME

**Figure 1:**
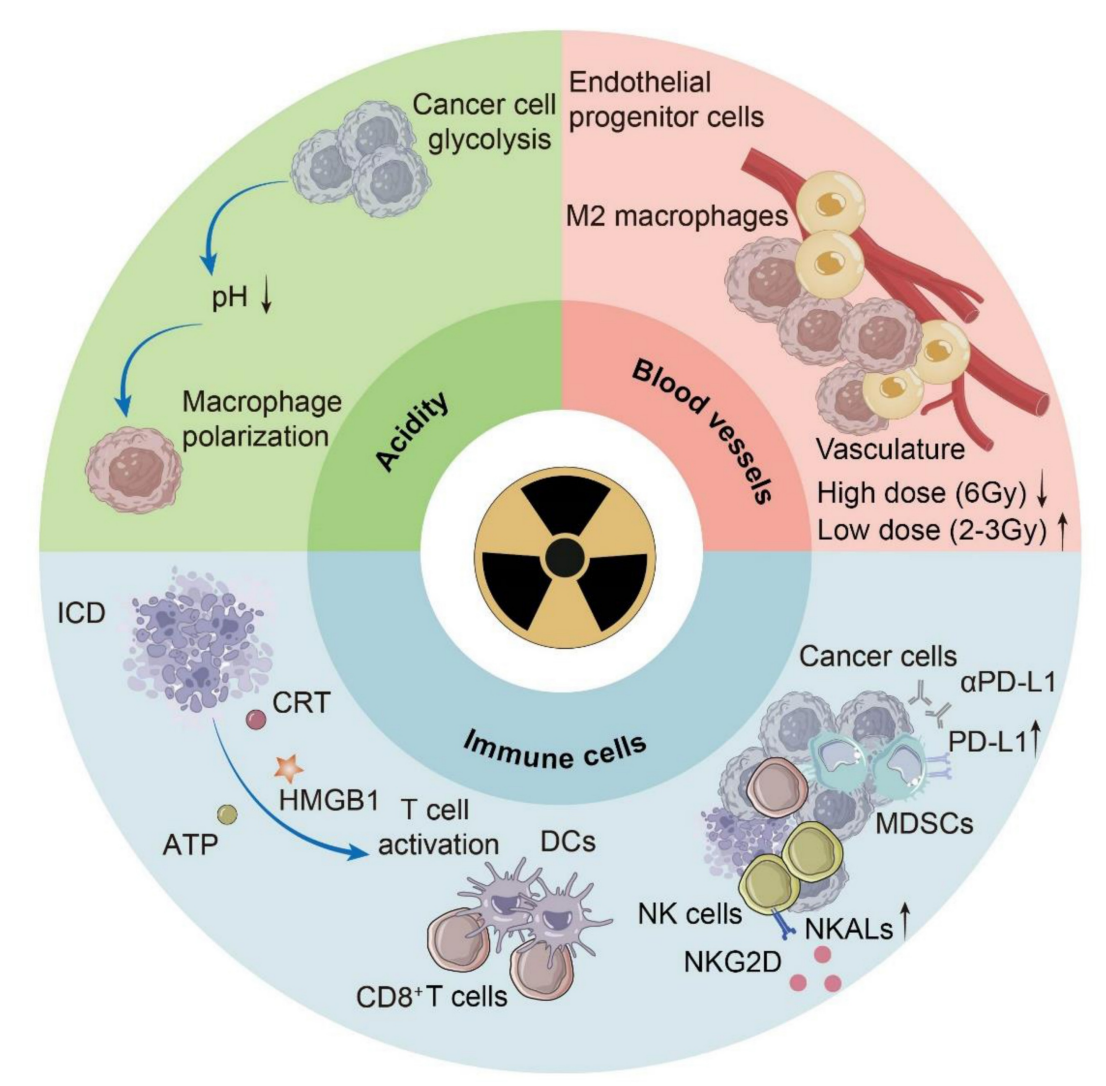
** Several changes in the tumor microenvironment (TME) occur during radiotherapy.** The acidity of the TME is attributed mainly to glycolysis in cancer cells and is sensed by macrophages, leading to macrophage polarization. ICD in tumors caused by radiotherapy releases DAMPs, including ATP, CRT and HMGB1, which help with the initiation of adaptive immunity. Radiotherapy has different effects on various types of immune cells, such as upregulating the expression of PD-L1 in MDSCs and increasing the number of NKALs specific for NKG2D. Radiotherapy also affects the pattern and efficiency of blood vessel development in a dose-dependent manner. ICD, immunogenic cell death; DAMPs, damage-associated molecular patterns; ATP, adenosine triphosphate; CRT, calreticulin; HMGB1, high-mobility group box-1 protein; DCs, dendritic cells; PD-L1, programmed death ligand 1; MDSCs, myeloid-derived suppressor cells; NKALs, NK cell-activating ligands; NKG2D, natural killer group 2 member D.

Radiotherapy can transform “cold” tumors into “hot” tumors to enhance therapeutic effects, recruiting more immune cells to infiltrate the TME, increasing the expression of biomarkers, and leading to the development of immunogenic tumors 13. These changes are predominantly exemplified in the TME, which includes cancer associated with immune cells, blood vessels and noncellular factors such as cytokines, chemokines and metabolites. Its characteristics include acidity, hypoxia, immune cell recruitment, and increased lactate and reduced glucose concentrations, which change with distance from blood vessels **(Fig. 1)**.

### 2.1 Radiotherapy calls for acidity reversion in the TME

Acidosis, a fundamental characteristic of the TME, is mostly attributed to glycolysis in tumor cells 14. Acidification of the TME has long been blamed for poor prognosis, which is related to increased invasion ability and immune escape 15. On the one hand, the acidic environment alters the expression of specific genes at the transcriptional level, contributing to tumor metastasis 15. On the other hand, macrophages act as the medium for the disability of T cells and irresponsiveness to immunotherapy 16. Conclusively, G protein-coupled receptors (GPCRs) on the surface of tumor-associated macrophages sense acidity, leading to an increase in cyclic adenosine monophosphate (cAMP), consequently increasing the expression of the transcriptional repressor inducible cAMP early repressor (ICER) and ultimately macrophage polarization toward a noninflammatory phenotype 16. Although our knowledge about the effect of radiotherapy on acidity in the TME is still limited, neutralization of tumor acidity has been shown to promote the antitumor effect of radiotherapy, and the reversion of tumor acidity is now a key consideration in the development of antitumor nanomedicines 17.

### 2.2 Radiotherapy modulates immune cells in the TME

The effects of radiotherapy on immune cells play a dominant role in the remodeling of the TME, including both immune activation and immune suppression. For immune activation, radiotherapy causes ICD in tumors, which is characterized by the release of endogenous adjuvants named damage-associated molecular patterns (DAMPs), and fuels the release of DAMPs by increasing ROS, endoplasmic reticulum (ER) stress and, consequently, the unfolded protein response (UPR) 18. DAMPs include surface-exposed calreticulin (CRT) and heat shock proteins (HSPs), which passively release high mobility group box-1 protein (HMGB1) and adenosine triphosphate (ATP), which are liberated into the extracellular space. Dendritic cells (DCs), as antigen-presenting cells, play a significant role in reacting to ICD and initiating adaptive immunity. CRT functions as an “eat me” signal to promote the uptake of dying tumor cells by DCs 19. HMGB1, which binds to pattern recognition receptors (PRRs), facilitates the maturation of DCs and the release of proinflammatory cytokines, especially interferon-I (IFN-I) 20. Tumor-infiltrating DCs play a major role in IFN-I induction *via* the cyclic GMP-AMP synthase (cGAS)-stimulator of interferon genes (STING) pathway due to the accumulation of mtDNA in the cytoplasm 21. ATP acts as a “find me” signal for DC precursors and macrophages to facilitate the recruitment of myeloid cells 22. Radiotherapy increases the number of tumor-associated macrophages (TAMs) and induces a phenotype transition from M2 to M1, which is proinflammatory 23. Additionally, radiotherapy increases major histocompatibility complex class-I (MHC-I) expression on tumor cells, promoting the infiltration of CD8^+^ T cells 24. It has been suggested that 12Gy radiation can significantly increase the secretion of C-X-C motif chemokine ligand 16 (CXCL16), which plays an important role in recruiting Th1 and cytotoxic T lymphocytes (CTLs) to all mouse and human breast cancer cells 25. Additionally, radiotherapy can increase the expression of NK cell-activating ligands (NKALs) specific for natural killer group 2 member D (NKG2D), enhancing the killing effect on tumor cells 26. In terms of immunosuppressive effects, radiation-induced lymphopenia (RIL) is reported as a potential adverse event, which is discussed later in this review 27. Prolonged stimulation by IFN-I may cause immune exhaustion 28. Additionally, radiotherapy helps with the mobilization of immunosuppressive cells. Radiotherapy increases C-C motif chemokine ligand 2 (CCL2) production, which facilitates C-C motif chemokine receptor 2 (CCR2)^+^ Treg recruitment 29. Radiation can promote the infiltration of myeloid-derived suppressor cells (MDSCs) *via* the CCL2-CCR2 pathway 30. Furthermore, radiotherapy can upregulate the expression of programmed death ligand 1 (PD-L1) in MDSCs, and combined treatment with PD-L1 blockade and immunogenic hypofractionation can help improve tumor immunity 31.

### 2.3 Radiotherapy modulates angiogenesis in the TME

In response to the demand for oxygen and nutrients for tumor growth and progression, blood vessels develop through either angiogenesis or vasculogenesis, which are responsible for hypoxia and acidosis in the TME 32. This process is inevitably affected as radiotherapy is adopted. Under radiation, the formation of blood vessels switches from sprouting angiogenesis to intussusceptive angiogenesis as an adaptive mechanism, similar to the response to antiangiogenic therapies 33. In general, radiotherapy damages endothelial cells to inhibit *de novo* and ongoing angiogenesis 34. Because vasculogenesis relies on angioblast differentiation rather than endothelial cells, it is vital for tumor growth under radiotherapy 32. The effect of radiotherapy on angiogenesis is time- and dose-dependent; high-dose (6 Gy) radiation causes a decrease in the tumor vasculature, whereas low-dose (2-3 Gy) radiation has a transient proangiogenic effect 35, 36. In addition to the effects of radiotherapy on angiogenesis, the abnormal vasculature in tumors is responsible for resistance to radiotherapy 37. As hypoxia exacerbates, the level of hypoxia-inducible factor-1α (HIF-1α) increases, increasing its downstream target stromal cell-derived factor-1 (SDF-1) and consequently recruiting proangiogenic macrophages as well as endothelial progenitor cells 38. Therefore, the use of angiogenesis inhibitors in a sophisticated manner for blood vessel normalization has been adopted as a strategy 39.

## 3. Radiotherapy modulates tumor metabolism

**Figure 2:**
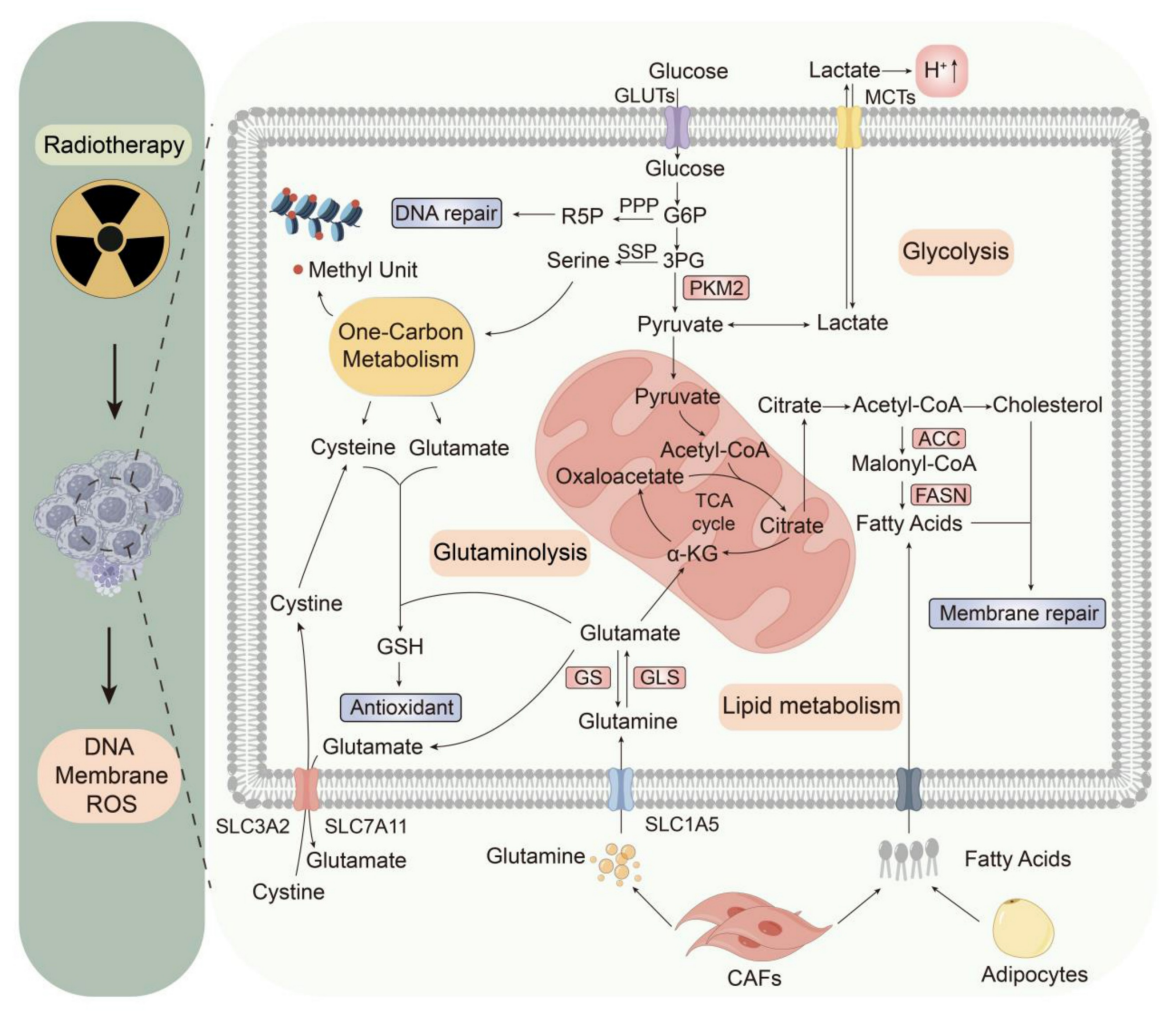
** Radiotherapy-mediated metabolic shifts within the TME.** Radiotherapy triggers tumor metabolic rewiring to enhance survival. Glycolysis fuels ATP, while PKM2 inactivation diverts intermediates to the PPP and SSP. Glutamine metabolism supports nucleotide and glutathione synthesis *via* GLS. Enhanced lipogenesis and lipid scavenging from stromal cells (*e.g.*, CAFs and adipocytes) aid membrane repair. Tumor-secreted ROS and lactate suppresses immune activity, while stromal cells supply nutrients, fostering therapy resistance. Abbreviations: PKM2, pyruvate kinase M2; PPP, pentose phosphate pathway; SSP, serine synthesis pathway; GLS, glutaminolysis; CAF, cancer-associated fibroblast; ROS, reactive oxygen species.

Radiotherapy can eliminate tumor cells primarily by ROS-induced DNA damage and, secondarily, by destroying cellular structures such as the cell membrane. Despite numerous advances over the past few decades, radioresistance remains a challenge in radiotherapy, which can be attributed to changes in metabolism and the TME, as well as their interplay 40. Therefore, understanding metabolic changes is particularly important for future radiotherapy, especially those related to energy utilization, redox balance, nucleic acid synthesis, and membrane repair **(Fig. 2)**.

### 3.1 Radiotherapy modulates glycolysis

Glucose is one of the main nutrients (the other being glutamine) that support the survival and proliferation of cancer cells. More than 70% of human cancers exhibit amplified glycolytic gene expression 41, a metabolic feature further exacerbated by radiotherapy-induced glycolysis upregulation linked to malignant phenotypes and treatment resistance 5. In the absence of radiation, most glucose is converted to pyruvate *via* lactate dehydrogenase (LDH). This process recycles nicotinamide adenine dinucleotide (NAD) to sustain glycolysis in cancer cells 42. However, a decrease in the activity of the M2 isoform of pyruvate kinase (PKM2) is accompanied by an increase in ROS caused by radiation, implying that limiting pyruvate synthesis may increase tumor cell tolerance to oxidative stress 43, 44. Paradoxically, despite reduced PKM2 enzymatic activity expected to decrease both pyruvate and lactate levels, the post-radiation lactate content significantly increases over time. This contradictory effect is presumably due to the oxidative decarboxylation of malate to pyruvate 45, increased expression of monocarboxylate transporter 1 (MCT1), which facilitates the export of lactate 46, and increased LDH activity in the anaerobic glycolytic state 44.

The inactivity of PKM2 reduces the synthesis of pyruvate and blocks glycolysis, which provides more upstream glycolytic intermediates to promote auxiliary glycolytic pathways related to the pentose phosphate pathway (PPP) and serine synthesis pathway (SSP) 47. The up-regulated PPP metabolism can regenerate nicotinamide adenine dinucleotide phosphate (NADPH) to combat oxidative stress caused by ROS, produce ribose 5-phosphate (R-5-P) to facilitate nucleic acid synthesis, and supply glycerol-3-phosphate for the synthesis of membrane phospholipids 48, 49. The overexpressed SSP metabolism contributes to NADPH generation, and *via* serine-driven one-carbon metabolism, it generates glutathione (GSH) precursors such as glycine and cysteine 50, 51. Serine promotes an interconnected metabolic pathway network for carbon metabolism, promoting the biosynthesis of nucleotides, S-adenosylmethionine (SAM), NADPH, and GSH. Even when tumor cells increase the synthesis of serine, they still absorb a large amount of serine from the environment 52.

Multiple drugs, such as glycolytic inhibitors and pyruvate dehydrogenase (PDH) modulators, have been designed to customize cancer cell metabolism. Despite failures in clinical trials due to metabolic heterogeneity and reprogramming of the TME, combining radiotherapy, metabolism, and immunotherapy may have unexpected effects. Emerging evidence highlights the regulatory roles of miRNAs in modulating radioresistance. Specifically, miR-200a is inversely correlated with the expression of DNA repair enzymes, whereas microRNA-200c enhances the radiosensitivity of human cancer cells by modulating prosurvival signaling pathways and suppressing epithelial‒mesenchymal transition (EMT) 53, 54. These findings underscore miRNAs as novel therapeutic targets to mitigate radioresistance, suggesting a promising strategy for combination therapy in cancer 55. Additionally, biocompatible liposomes loaded with mannose and levamisole hydrochloride have been designed to regulate glucose and mitochondrial metabolic pathways in tumor cells and macrophages simultaneously. When combined with radiotherapy, this drug reverses the immunosuppressive TME by inhibiting M2 macrophages and further induces effective ICD and a systemic antitumor immune response 56.

### 3.2 Radiotherapy modulates glutaminolysis

Glutamine, the second major nutrient for tumor cells, is the most abundant free amino acid and plays an important role in tumor cell biology. In addition to being hallmarks of fast glucose metabolism, many types of tumors are characterized by elevated glutamine consumption 57. Under normal circumstances, glutamine uptake is strongly regulated in cells. However, tumor cells require large amounts of glutamine and overcome this uptake and usage limitation by inducing the expression of glutamine transporters, glutamine synthetase (GS) and glutaminase (GLS) after radiation 44, 58, 59. In the context of glutamine or glucose depletion, tumor cells might increase glutaminolysis to ensure the perpetuation of the tricarboxylic acid (TCA) cycle by stimulating α-ketoglutarate (α-KG) synthesis from glutamate, the end-product of glutaminolysis 60.

In addition to maintaining the TCA cycle, glutamine metabolism serves a multitude of pivotal physiological functions. Glutamine is an important precursor that contributes to purine and pyrimidine synthesis 61. Because radiation damages DNA, tumor cells need to increase DNA synthesis to aid in repair. GS, which is transcriptionally regulated by signal transducer and activator of transcription 5 (STAT5) in response to radiation, facilitates nucleotide metabolism and subsequent DNA repair 62. This damage is not fatal to some extent, but the disadvantages of the capacity for DNA repair and the efficiency of radiation damage cause cell death 63. Interestingly, the decomposition of glutamine, the raw material of GSH, is postulated to regulate ferroptosis through the provision of α-KG in the TCA cycle 64. However, high glutamine metabolic flux is converted into α-KG and enters the TCA cycle, which leads to the accumulation of a large amount of ROS, resulting in reduced expression of SLC7A11 and reduced synthesis of GSH after ionizing radiation. This results in oxidative stress injury to cells and ultimately leads to cell death 65. Recent studies have shown that the inhibition of glutamine facilitates immunogenic tumor ferroptosis, further increasing the effectiveness of radiotherapy 8. Considering that glutamine depletion has great adverse effects on the human body, the effect of glutamine supplementation in the tumor-bearing state needs further study.

### 3.3 Radiotherapy modulates lipid metabolism

Radiation not only damages DNA but also causes damage to the cell membrane. In addition to considerable changes in glucose and glutamine metabolism, lipid metabolism is also significantly altered in tumor cells to repair the cell membrane and meet energy demands for proliferation 59. As mentioned earlier, the process of glycolysis provides more upstream glycolytic intermediates to promote auxiliary glycolytic pathways reelected in the PPP and SSP after radiation, and this regulation of the PPP provides more NADPH necessary for lipid synthesis. Fatty acids are integral components of cell membranes and are crucial for repairing damage caused by radiation exposure.

Tumor cells fulfill their requirements for fatty acids through two primary mechanisms: *de novo* synthesis *via* the enzymatic actions of acetyl-CoA carboxylase (ACC) and fatty acid synthase (FASN), as well as the uptake of lipids from the TME. Current research indicates that FASN overexpression in tumors is associated with radioresistance 66. Uniformly, previous studies have shown that inhibiting fatty acid biosynthesis results in increased cytotoxicity and sensitivity to irradiation with FASN siRNA 59. Mechanistically, FASN confers radioresistance *via* the Akt- and NF-κB-mediated signaling pathways because the levels of proteins upstream of FASN, such as Akt and NF-κB, are increased in radioresistant tumor cells 66. Additionally, cholesterol is involved in cell membrane synthesis and angiogenesis, and dysregulation of cholesterol metabolism has been linked to radiation resistance, suggesting a potential target for enhancing the efficacy of radiotherapy 67.

Several studies have demonstrated that the TME can supply lipids to tumor cells, thereby facilitating their survival following radiotherapy 68. Cancer-associated fibroblasts (CAFs) associated with pancreatic and colorectal cancers are known to secrete lipids, whereas tumor cells can induce the decomposition of fat in adipocytes 69. Hypoxia, which is a common consequence of radiation therapy, can suppress the oxygen-dependent pathway of *de novo* lipid synthesis in cancer cells, leading to increased reliance on exogenous lipid uptake 70. By enhancing the absorption and accumulation of fatty acids, tumor cells can develop resistance to ROS, thus promoting their survival.

### 3.4 Crosstalk between metabolism and cell death

Cell death may occur under various stress conditions, such as hypoxia, nutrient deprivation, and external stimuli 71. In the hypoxic TME, the phosphatidylinositol 3-kinase (PI3K)/Akt/HIF-1α signaling axis is activated, which in turn modulates the glycolytic process 72. The high rate of glycolysis in tumor cells leads to substantial lactate production, thereby creating an acidic TME that is inherently immunosuppressive 73. Concurrently, the metabolite α-KG recruits procaspase-8 and gasdermin C (GSDMC), leading to the activation of pyroptosis in an acidic environment 74. Apoptosis 75, necrosis 76, and pyroptosis 77 are linked to energy stress, suggesting that energy stress could be a major trigger of cell death. AMP-activated protein kinase (AMPK), a cell death regulator, also functions as a crucial intracellular energy sensor, playing a significant role in the coordination of metabolism, cell death, and inflammatory responses 71. Upon sensing an elevated ADP/ATP ratio, AMPK activates downstream signaling pathways, facilitating energy generation 78. Additionally, lipid metabolism is associated with apoptosis 79, pyroptosis 80, and ferroptosis 81. In particular, the metabolites, key enzymes and transcription factors involved in lipid metabolism participate in the regulation of ferroptosis. Ferroptosis is closely related to lipid peroxidation, and glutaminolysis plays a crucial role in the death process 64. The nexus between cell death induced by radiotherapy and cellular metabolism has not been fully elucidated. Future studies are warranted to explore the interplay between metabolic pathways and models of cell death within the TME.

## 4. Radiotherapy induces ICD

**Figure 3:**
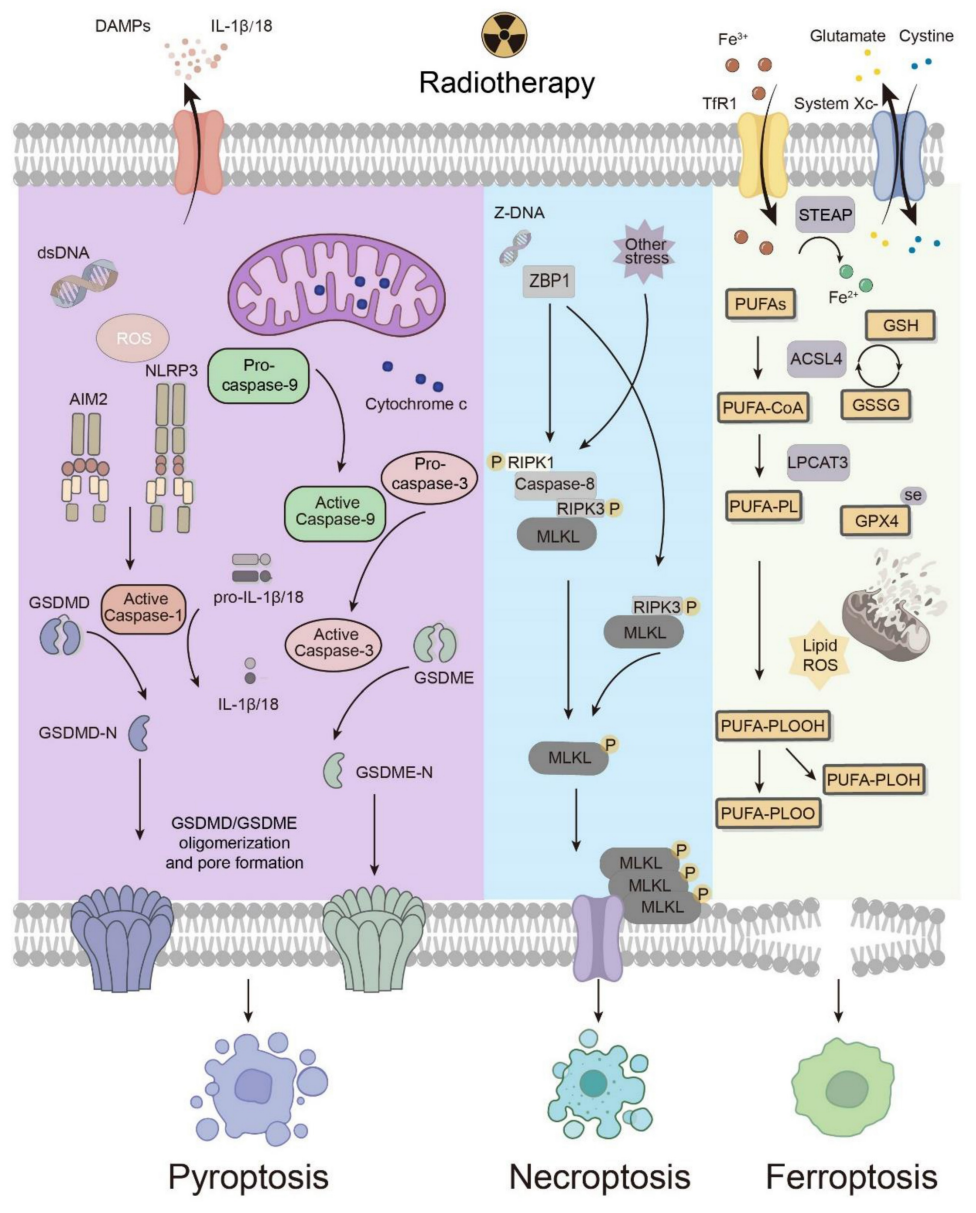
** Mechanisms of immunogenic cell death modalities under radiotherapy.** The types of pyroptosis induced by radiotherapy include the inflammasome-caspase 1-GSDMD pathway and the caspase 3-GSDME pathway. Necroptosis elicited by radiotherapy mainly relies on a ZBP1-mediated pathway. Ferroptosis, which involves the executive and suppressive systems, is driven by lipid peroxidation, which can be facilitated by radiotherapy and the consequential increase in ROS. ACSL4 and GPX4 are known targets of radiotherapy. DAMPs, damage-associated molecular patterns; ROS, reactive oxygen species; AIM2, absent in melanoma 2; NLRP3, NOD-, LRR- and pyrin domain-containing protein 3; ZBP1, Z-DNA-binding protein 1; RIPK1, receptor-interacting protein kinase 1; RIPK3, receptor-interacting protein kinase 3; MLKL, mixed lineage kinase domain-like protein; TfR1, transferrin receptor 1; STEAP, six-transmembrane epithelial antigen of prostate; PUFA-PL, polyunsaturated fatty acid-containing phospholipid; ACSL4, acyl-CoA synthetase long chain family member 4; LPCAT3, lysophosphatidylcholine acyltransferase 3; GPX4, glutathione peroxidase 4; GSH, glutathione; GSSG, oxidized glutathione.

Radiotherapy elicits several modalities of cell death, among which pyroptosis, necroptosis and ferroptosis are the most common and well studied. Based on these molecular pathways, key points related to the response to irradiation have been identified, revealing the mechanisms of regulated cell death induced by radiotherapy **(Fig. 3)**. Here, how pyroptosis, necroptosis and ferroptosis interact with antitumor immunity differently is also summarized and discussed.

### 4.1 Pyroptosis

Pyroptosis, characterized by the activation of inflammatory caspases and proinflammatory cytokines as well as pore formation by gasdermins (GSDMs) on the plasma membrane, is a double-edged sword in antitumor immunity under radiotherapy 9. The release of proinflammatory cytokines may favor tumorigenesis, whereas the immunogenic property of pyroptosis initiates adaptive immune responses to eliminate tumors. Pyroptosis can be evoked *via* parallel pathways, each of which is characterized by one of the GSDMs, including GSDMA, GSDMB, GSDMC, GSDMD, GSDME, and DFNB59 10. The N-terminus, which is exposed due to the cleavage of GSDMs, forms pores on the cell membrane, disrupting the integrity of the membrane 82. Consequently, the cell contents flow out, and proinflammatory cytokines are released, leading to inflammatory cell death. Despite sharing this generally common mechanism, only two of these pathways, namely, the inflammasome-caspase 1-GSDMD pathway and the caspase 3-GSDME pathway, have been verified to be involved in how radiotherapy induces pyroptosis. To better understand how these cascades work under radiotherapy and identify potential targets for medical development, it is necessary to elucidate the key molecules involved.

The inflammasome-caspase 1-GSDMD pathway is considered the canonical pathway of pyroptosis, and its mechanism has been revealed at the molecular level. When the PRR is stimulated, the inflammasome produces mature caspase 1, which cleaves GSDMD into N- and C-terminal domains. Simultaneously, caspase 1 transforms the precursors of IL-1β and IL-18 into their activated forms 9. Notably, NOD-, LRR- and pyrin domain-containing protein 3 (NLRP3) and absent in melanoma 2 (AIM2) have been shown to be stimulated by irradiation and then trigger the downstream pathway, leading to cell death. DNA damage and cell death caused by irradiation lead to an increase in dsDNA, which activates AIM2 inflammasomes 83. Additionally, irradiation promotes the production of mitochondrial reactive oxygen species (mROS), which are regulated by Ca^2+^ influx and K^+^ efflux, activating the NLRP3 inflammasome 84. Impressively, not only can the cleavage of GSDMD be facilitated *via* the activation of NLRP3 and AIM2 by radiotherapy, but the expression of GSDMD itself can also be upregulated by irradiation 85. Caspase 1, which activates IL-1 and IL-18, can also be activated directly by radiotherapy 86. IL-18 is dispensable for antitumor effects, whereas the IL-1 pathway in DCs facilitates the survival and activation of CD8^+^ T cells under irradiation through cross-priming 87.

With respect to the caspase 3-GSDME pathway, although its subsequent influence on antitumor immunity remains limited, the choice between pyroptosis and apoptosis requires further exploration 88. The caspase 3-GSDME pathway is triggered by radiotherapy *via* the production of ROS, which leads to the oxidation and aggregation of Tom20 89. Oxidized Tom20 translocates Bax to mitochondria, promoting the release of cytochrome c from the mitochondria into the cytoplasm, which activates caspase 9 and then consequently activates caspase 3 89. When GSDME is expressed at a high level, caspase 3 cleaves GSDME, inducing pyroptosis; when GSDME is expressed at a low level, caspase 3 induces apoptosis 88. Intriguingly, irradiation itself can promote the expression of GSDME in several tumor cell lines 90. Overall, radiotherapy induces pyroptosis in high GSDME-expressing tumor cells, and hyperfractionated radiotherapy with specific chemotherapeutic agents potentially induces pyroptosis more extensively.

### 4.2 Necroptosis

Necroptosis, a proinflammatory and immunogenic modality of regulated cell death, is characterized by receptor-interacting protein kinase 3 (RIPK3)-dependent mixed lineage kinase domain-like protein (MLKL) phosphorylation 9. Mechanically, upon extracellular or intracellular stress initiating death receptors, the state of receptor-interacting protein kinase 1 (RIPK1), ubiquitination or phosphorylation, determines the fate of a cell at the crossroads of survival and death 91. Its interaction with caspase 8 and RIPK3 plays a critical role in leading the cell to undergo apoptosis or necroptosis, as caspase 8 enables apoptosis while inhibiting RIPK3-MLKL-mediated necroptosis 92. In RIPK1-dependent or RIPK1-independent manners, phosphorylated RIPK3 activates MLKL, which is the primary executor of necroptosis. Phosphorylation of MLKL induces oligomerization and translocation of MLKL to the cell membrane, followed by cell membrane permeabilization, subsequently leading to pore formation and the release of DAMPs 93. This immunogenic consequence, along with RIPK1 and NF-κB signaling, gives rise to cross-priming of CD8^+^ T cells, facilitating adaptive immunity 94.

Although RIPK1, RIPK3 and MLKL were discovered successively over a decade ago, necroptosis in tumors subjected to radiotherapy remained elusive until the Z-DNA-binding protein 1 (ZBP1)-mediated pathway was first described 95. The activation of ZBP1 has been reported to initiate RIPK3-mediated MLKL activation, which is RIPK1 independent.

Immunologically, ZBP1-mediated necroptosis has been reported to interact with the cGAS-STING pathway, which results in the activation of innate immunity 96. The cGAS-STING pathway is also activated by cytosolic DNA to initiate innate immunity. In the TME after radiation treatment, mtDNA is an emerging key to activating the cGAS‒STING pathway, and the ZBP1‒RIPK3‒MLKL cascade induces the release of mtDNA from mitochondria and leads to the accumulation of cytosolic mtDNA. In addition to the ZBP1-mediated cascade, elevated levels of mitochondrial ROS, which can be induced by radiotherapy, may spark off RIPK1-RIPK3-MLKL-mediated necroptosis 97. Collectively, necroptosis, especially ZBP1-mediated necroptosis, contributes to both the innate and adaptive immune systems for postradiotherapy antitumor immunity.

### 4.3 Ferroptosis

Ferroptosis is an iron-dependent form of regulated cell death driven by lipid peroxidation 12, 98. The molecular mechanism of ferroptosis can be summarized as suppressive systems, which include the glutathione peroxidase 4 (GPX4)-dependent pathway as well as GPX4-independent pathways, and the executive system, which relies on the synthesis of polyunsaturated fatty acid-containing phospholipids (PUFA-PLs) along with sufficient ROS and ferrous ions to initiate lipid peroxidation 99. Several sections of the suppressive and executive systems can act as targets under irradiation to trigger ferroptosis.

In the executive system of ferroptosis, the biosynthesis of PUFA-PLs lays a foundation for lipid peroxidation. The peroxidation and accumulation of PUFA-PLs trigger the collapse of the cell membrane, leading to cell death 100. With acyl-CoA synthetase long chain family member 4 (ACSL4) as a catalyst, long-chain PUFAs are ligated with CoA, whereupon PUFA-PLs are generated by lysophosphatidylcholine acyltransferase 3 (LPCAT3) 101. Fe^3+^ enters the cytoplasm *via* transferrin receptor 1 (TfR1) and is reduced to Fe^2+^ by six-transmembrane epithelial antigen of the prostate (STEAP) 64. The degradation of heme mediated by heme oxygenase-1 (HO-1) provides divalent iron as well 99. Iron is involved in not only lipid peroxidation catalyzed by lipoxygenase (LOX) and cytochrome P450 oxidoreductase (POR) but also the Fenton reaction: PLOOHs react with ferric and ferrous ions to generate free radicals, which subsequently react with PUFA-PLs to multiply PLOOH 102. Additionally, as iron is a component of the electron transport chain, iron homeostasis is closely related to the production of mitochondrial ROS. On the one hand, radiotherapy upregulates ACSL4 to facilitate PUFA-PL biosynthesis 99. On the other hand, radiotherapy promotes iron release from heme and ferritin 99. Moreover, irradiation damages the structure and function of mitochondria, increasing the production of reactive oxygen species (ROS), which are also key factors in the executive system of ferroptosis 99.

In terms of the suppressive systems of ferroptosis, the GPX4-dependent system, also known as the System Xc^-^-GSH-GPX4 signaling axis, is prominent. System Xc^-^ consists of SLC3A2 and SLC7A11, which export intracellular glutamate while importing extracellular cystine, which is later reduced to cysteine and subsequently serves as an ingredient in the synthesis of GSH 103. GSH not only eliminates intracellular ROS but also provides electrons when GPX4 catalyzes the reduction of PLOOH, which elucidates how GPX4 disrupts the accumulation of lipid peroxides and consequently suppresses ferroptosis 99. Radiation has been shown to lead to GSH depletion, which favors ferroptosis by weakening GPX4-mediated suppression 99. Lei *et al.* reported that irradiation induces the expression of SLC7A11 and GPX4, inhibiting ferroptosis, as an adaptive response to the upregulation of ACSL4 99.

In the TME, ferroptosis of tumor cells and various immune cells facilitates or inhibits overall antitumor immunity and the functions of each other. Recent research has proposed that cancer cell death from ferroptosis impedes the antigen-presenting function of DCs, which may be related to the earlier finding that several kinds of oxidized lipids can hinder the activation of CTLs through blocking antigen presentation by DCs 104. Moreover, the maturation and function of DCs in the microenvironment can also be negatively impacted by the death of DCs from ferroptosis 105. Similarly, polymorphonuclear myeloid-derived suppressor cells (PMN-MDSCs) reportedly impair the antigen-presentation function of DCs *via* myeloperoxidase-driven lipid peroxidation and accumulation 106. Additionally, in the TME, PMN-MDSCs that die spontaneously from ferroptosis release oxygenated lipids, which limits the activity of T cells and induces an immunosuppressive TME. Subsequent experiments demonstrated that inhibition of ferroptosis can abolish the suppressive effect of PMN-MDSCs and synergize with immune checkpoint blockade to repress tumor progression 107. CTLs release IFN-γ, which favors ferroptosis in tumor cells 108. Overall, ferroptosis in tumor cells and intratumoral immune cells still needs further exploration, with a focus on the precise induction of ferroptosis and the maximized effect of immunotherapy.

## 5. Radiotherapy Combined with Immunotherapy

Currently, radiotherapy combined with immunotherapy has been widely employed clinically, and various clinical trials have made progress in optimizing radiation modalities and immunotherapy targets. However, because adverse events of radiotherapy may impair immunity and overall well-being, appropriate management can be important for positive outcomes.

### 5.1 Clinical advancements

Radiotherapy combined with immunotherapy has demonstrated significant potential in enhancing antitumor immune responses and inducing abscopal effects. First, combination therapy facilitates the infiltration and activation of immune-stimulating cells induced by radiotherapy 109. Concurrently, radiotherapy overcomes immune tolerance, thereby increasing the clinical efficacy of ICB 110, 111. Second, abscopal effects are rare with monotherapy, particularly in tumors with low immunogenicity. However, the abscopal effects increase when radiotherapy is combined with immunotherapy 112. For example, a phase II trial (NCT02474186) in metastatic solid tumors reported that radiotherapy combined with granulocyte‒macrophage colony‒stimulating factor (GM-CSF) elicited abscopal responses in 26.8% of patients, with prolonged median overall survival. This review summarizes recent clinical trials that have investigated diverse combinatorial strategies pairing distinct radiation modalities with immunotherapeutic agents, aiming to amplify the immunostimulatory effects of RT while minimizing adverse outcomes **(Table 1)**. Nevertheless, the therapeutic benefits remain constrained by tumor heterogeneity, immunosuppressive microenvironments, and interpatient variability. Future research should prioritize the optimization of radiation dose fractionation regimens, the identification of predictive biomarkers, and the exploration of novel immunomodulators to overcome current limitations and maximize synergistic potential. Besides, synergistic effects between radiotherapy and other antitumor therapies, like tumor treating fields (TTFields) for glioblastoma treatment, are also worth exploring for their clinical prospects 113, 114.

### 5.2 Adverse events and management

Although advancements in the combination of radiotherapy and immunotherapy are promising, the risks associated with radiotherapy should not be neglected, especially impairment of the immune system 115. Adverse events caused by radiotherapy can be categorized into stochastic effects and tissue reactions. Stochastic effects refer to DNA mutations triggered by radiation, leading to secondary malignant neoplasms (SMNs). Tissue reactions, or deterministic effects, involve tissue and organ dysfunction attributed to cell death. This category can be further divided into short-term adverse effects, which occur during radiotherapy or within 3 months after radiotherapy, and long-term consequences, which are observed thereafter 116. The former, such as mucositis and diarrhea, heal within weeks to months, whereas the latter can be irreversible and progressive. Therefore, RIL is worthy of extra attention 27. RIL has been reported in various types of solid tumors, such as head and neck cancer, lung cancer and prostate cancer, and is associated with poor prognosis 117-119. Given that a decreased lymphocyte count after radiotherapy has been proven to result in unsatisfactory efficacy of ICB, protection of the immune system should be emphasized when combining radiotherapy with immunotherapy 120.

To maximize patients' benefit from radiotherapy, measures need to be taken before, during and after treatment to prevent and address adverse events 115. Generally, conversations should be conducted with patients before treatment to inform them of potential risks and achieve good patient compliance, including nutritional support as well as smoking and alcohol cessation. During the course of treatment, dose adaptation, scheduling and fractionation should be performed when necessary, according to monitoring of patients' responses. After treatment, follow-up inspection visits and interventions are important to resolve undesirable symptoms secondary to the treatment. In particular, extensive exploration has been conducted to prevent RIL. Because the severity of RIL is associated with the baseline lymphocyte count, screening patients with high RIL risk before radiotherapy can be meaningful 117. In addition, the adoption of hypofractionated radiotherapy can minimize RIL 121. Another focus in this area is the prevention of SMNs. Given that stochastic effects do not occur with a specific dose threshold, the management of radiation dose and fractionation may be inadequate to mitigate the pro-oncogenic effect 122. Strategies have been developed to protect DNA from radiation damage and are also supposed to be normal-tissue selective so as not to impede the tumor-killing effect. For example, the clinically approved amifostine can be employed as both a topical and systemic strategy by scavenging radiation-induced free radicals, providing DNA protection and accelerating repair 123. In addition, antioxidants, including tempol and manganese superoxide dismutase (MnSOD), are used to attenuate radiation-induced oxidative stress 124, 125. Moreover, inhibitors of SRC family kinases (SFKs), which are crucial in the formation of the radiation-induced pro-tumorigenic microenvironment, have been employed recently 126. SFK inhibitors have improved radiotherapy-elicited antitumor effects, indicating the potential of reshaping the microenvironment to prevent radiation-induced carcinogenesis.

## 6. Conclusions and Perspectives

This review focuses on the radiotherapy-induced TME, metabolic alterations, ICD and consequent changes in antitumor immunity. The radiation-induced effects on cell metabolic pathways and the mechanisms of pyroptosis, necroptosis and ferroptosis suggest potential targets that may be utilized in medical development to synergize with radiotherapy and immunotherapy. Investigating the crosstalk between cellular metabolism and cell death in the TME may help in understanding this process. However, the underlying relationship between radiation-induced alterations in metabolism and radiation-induced cell death requires further exploration. To date, ICD, especially ferroptosis, has been the intersection of radiotherapy and immunotherapy, playing a significant role in nanomedicines. Metabolic drugs on a postradiotherapy basis are expected to interact with immunology more in antitumor strategies in the future.

The revealed and unknown mechanisms of ICD modalities encapsulate potential targets for antitumor therapies and may contribute to connecting metabolism with cell death. Ferroptosis, as discussed above, plays a crucial role in radiotherapy-induced cell death and tumor suppression, mediating the synergy between radiotherapy and immunotherapy. With increasing knowledge about cell death and cellular metabolism, new medicines may be developed to combine radiotherapy and immunotherapy, maximizing the antitumor effects of multiple strategies.

The significant improvements in therapeutic effects indicate the exciting potential of the combination of radiotherapy and immunotherapy. Clinical trials conducted with different radiotherapy modalities have demonstrated encouraging synergistic effects with immunotherapies that have distinct targets. The expanded range of cancer types in clinical trials implies the broader use of radiotherapy combined with immunotherapy in the future. In addition, increased abscopal effects have been observed under this combined therapy, which may inspire subsequent attempts of this kind. In the future, the synergistic effects of radiotherapy and immunotherapy will play an increasingly important role in cancer treatment, with the potential to cure more patients with locally advanced tumors and to provide benefits beyond palliative care for patients with metastatic cancer.

## Figures and Tables

**Table 1:** Summary of clinical trials investigating the combination of radiotherapy and immunotherapy

Radiation	Drug	Targets	Type of cancer	Clinical trials	Phase	Status
RT	Pembrolizumab	PD-1	Head and neck cancer	NCT03383094	Phase 2	Recruiting
SBRT	Relatlimab, Nivolumab	LAG-3, PD-1	Uveal melanoma	NCT05077280	Phase 2	Recruiting
SBRT	CBI	CTLA-4, PD-1/PD-L1	Metastatic cancer	NCT02843165	Phase 2	Active, not recruiting
IMRT	Nivolumab	PD-1	Stage III non-small cell lung cancer	NCT04577638	Phase 2	Completed
SBRT	Gemcitabine, Penpulimab	PD-1	Osteosarcoma	NCT06114225	Phase 2	Recruiting
SBRT	Atezolizumab	PD-L1	Recurrent, persistent, or metastatic cervical cancer	NCT03614949	Phase 2	Recruiting
CIRT	Pembrolizumab	PD-1	Solid tumors	NCT05229614	Phase 2	Recruiting
RT	Atezolizumab	PD-L1	Penile cancer	NCT03686332	Phase 2	Completed
EBRT	Tremelimumab	CTLA-4	Bladder cancer	NCT03601455	Phase 2	Active, not recruiting
RT	Bevacizumab, Tislelizumab	VEGF, PD-1	Stage III non-small cell lung cancer	NCT05468242	Phase 2	Recruiting
Brachytherapy	TSR-042	PD-1	Endometrial cancer	NCT03955978	Phase 1	Active, not recruiting
RT	Bevacizumab	VEGF	Rectal cancer	NCT00113230	Phase 2	Completed
SBRT	Fresolimumab	TGF-β	Non-small cell lung cancer	NCT02581787	Phase 1 Phase 2	Completed
RT	Pembrolizumab, Axatilimab	PD-1, CSF-1R	Triple-negative breast cancer	NCT05491226	Phase 2	Active, not recruiting
TDLN-sparing RT	CBI	PD-1	Esophageal squamous cell carcinoma	NCT06676449	Phase 3	Recruiting
RT	BMS-986205	IDO1	Glioblastoma	NCT04047706	Phase 1	Active, not recruiting
RT	CDX-1140	CD-40	Unresectable and metastatic solid tumors	NCT04616248	Phase 1	Recruiting
SABR	Atezolizumab	PD-1	Metastatic tumors	NCT02992912	Phase 2	Active, not recruiting
SBRT	Nivolumab, Pembrolizumab	PD-1	Metastatic non-small cell lung cancer	NCT03825510	Phase 2	Completed
RT	SD-101	TLR9	Low-grade B-cell non-Hodgkin lymphomas	NCT03410901	Phase 1	Recruiting
RT	SD-101	TLR9	Solid tumors and lymphoma	NCT03322384	Phase 1 Phase 2	Completed
RT	SD-101	TLR9	Low-grade follicular lymphoma	NCT02927964	Phase 1 Phase 2	Completed
RT	Nivolumab, SD-101	PD-1, TLR9	Metastatic pancreatic cancer	NCT04050085	Phase 1	Completed
SBRT	Pembrolizumab, SD-101	PD-1, TLR9	Prostate cancer	NCT03007732	Phase 2	Active, not recruiting
SBRT	Sintilimab	PD-1	Hepatocellular carcinoma	NCT06313190	Phase 2	Recruiting
RT	Rituximab, Pembrolizumab	CD20, PD-1	Follicular lymphoma	NCT02677155	Phase 2	Completed
RT	Pembrolizumab	PD-1	Vulvar cancer	NCT04430699	Phase 2	Recruiting
SBRT	CDX-301	FLT3	Non-small cell lung cancer	NCT02839265	Phase 2	Completed
RT	CDX-301, CDX-1140	FLT3, CD40	Solid tumors	NCT04616248	Phase 1	Recruiting
RT	Toripalimab	PD-1	Laryngeal and hypopharyngeal cancer	NCT06039631	Phase 1	Recruiting
RT	GM-CSF	GM-CSF	Solid metastatic tumors	NCT02474186	Phase 2	Completed
RT	GM-CSF, PD1 antibody	GM-CSF, PD-1	Head and neck cancer	NCT05760196	Phase 2	Recruiting
RT	GM-CSF	GM-CSF	Thymoma	NCT05407649	Phase 2	Completed

RT, radiation therapy; SBRT, stereotactic body radiation therapy; IMRT, intensity-modulated radiation therapy; CIRT, carbon ion radiotherapy; EBRT, external beam radiation therapy; TDLN-sparing RT, tumor draining lymph node-sparing radiotherapy; SABR, stereotactic ablative radiotherapy; CBI, checkpoint blockaded immunotherapy.

## Abbreviations

TME: tumor microenvironment
ICB: immune checkpoint blockade
ROS: reactive oxygen species
GPCRs: G protein-coupled receptors
cAMP: cyclic adenosine monophosphate
ICER: inducible cAMP early repressor
ICD: immunogenic cell death
DAMPs: damage-associated molecular patterns
ER: endoplasmic reticulum
UPR: unfolded protein response
CRT: calreticulin
HSPs: heat shock proteins
HMGB1: high-mobility group box-1 protein
ATP: adenosine triphosphate
DCs: dendritic cells
PRRs: pattern recognition receptors
IFN-I: interferon-I
cGAS: cyclic GMP-AMP synthase
STING: stimulator of interferon genes
TAMs: tumor-associated macrophages
MHC-I: major histocompatibility class-I
CXCL16: C-X-C motif chemokine ligand 16
CTL: cytotoxic T lymphocyte
NKALs: NK cell-activating ligands
NKG2D: natural killer group 2 member D
RIL: radiation-induced lymphopenia
CCL2: C-C motif chemokine ligand 2
CCR2: C-C motif chemokine receptor 2
PD-L1: programmed death ligand 1
MDSCs: myeloid-derived suppressor cells
HIF-1α: hypoxia-inducible factor-1α
SDF-1: stromal cell-derived factor-1
LDH: lactate dehydrogenase
NAD: nicotinamide adenine dinucleotide
PKM2: M2 isoform of pyruvate kinase
MCT1: monocarboxylate transporter 1
PPP: pentose phosphate pathway
SSP: serine synthesis pathway
NADPH: nicotinamide adenine dinucleotide phosphate
R-5-P: ribose 5-phosphate
GSH: glutathione
SAM: S-adenosylmethionine
PDH: pyruvate dehydrogenase
EMT: epithelial-mesenchymal transition
GS: glutamine synthetase
GLS: glutaminase
TCA: tricarboxylic acid cycle
α-KG: α-ketoglutarate
STAT5: signal transducer and activator of transcription 5
ACC: acetyl-CoA carboxylase
FASN: fatty acid synthase
CAFs: cancer-associated fibroblasts
PI3K: phosphatidylinositol 3-kinase
GSDMC: gasdermin C
AMPK: AMP-activated protein kinase
GSDMs: gasdermins
NLRP3: NOD-, LRR- and pyrin domain-containing protein 3
AIM2: absent in melanoma 2
mROS: mitochondrial reactive oxygen species
RIPK3: receptor-interacting protein kinase 3
MLKL: mixed lineage kinase domain-like protein
RIPK1: receptor-interacting protein kinase 1
ZBP1: Z-DNA-binding protein 1
GPX4: glutathione peroxidase 4
PUFA-PL: polyunsaturated fatty acid-containing phospholipid
ACSL4: acyl-CoA synthetase long chain family member 4
LPCAT3: lysophosphatidylcholine acyltransferase 3
TfR1: transferrin receptor 1
STEAP: six-transmembrane epithelial antigen of prostate
HO-1: heme oxygenase-1
LOX: lipoxygenase
POR: cytochrome P450 oxidoreductase
PMN-MDSCs: polymorphonuclear myeloid-derived suppressor cells
GM-CSF: granulocyte-macrophage colony-stimulating factor
TTFields: tumor treating fields
SMNs: secondary malignant neoplasms
MnSOD: manganese superoxide dismutase
SFKs: SRC family kinases
